# Real-time tracking of vaccinia virus entry using a split luciferase complementation assay

**DOI:** 10.1128/jvi.01115-26

**Published:** 2026-09-29

**Authors:** Yunliang Zhang, Jaroslav Holly, James Gibbs, Jonathan W. Yewdell, Bernard Moss

**Affiliations:** 1 Laboratory of Viral Diseases, National Institute of Allergy and Infectious Diseases 35037, Bethesda, Maryland, USA; Northwestern University Feinberg School of Medicine, Chicago, Illinois, USA

**Keywords:** poxvirus, vaccinia virus, virus entry, endosome, membrane fusion, luminescence assay

## Abstract

A recombinant vaccinia virus (vA4-HiBiT) was constructed with a short, split luciferase HiBiT tag at the C-terminus of the A4 core protein open reading frame. When HeLa or A549 cells stably expressing LgBiT were infected with vA4-HiBiT, the interaction of the split luciferase fragments reconstituted NanoLuc enzyme activity. Upon addition of a membrane-permeable luciferase substrate, a strong luminescence signal indicated core entry into the cytoplasm within 5 min after infection and was proportional to virus multiplicity within the range of 0.5–80 PFU/cell. This direct entry assay was dependent on a functional virus entry–fusion complex, was inhibited by a neutralizing antibody, and was unaffected by an inhibitor of protein synthesis. Entry was enhanced by brief pH 5 treatment of cell-bound virus relative to pH 7 and prevented by pH 3 treatment. Bafilomycin A1, an inhibitor of endosomal acidification, exhibited greater inhibition of entry in A549-LgBiT cells compared to HeLa-LgBiT cells, as did treatment with ammonium chloride, suggesting variations in entry pathways or cell metabolism. Inhibition of entry by latrunculin A and cytochalasin D indicated a role for actin polymerization in both cell lines. Despite the discovery of a conserved furin cleavage consensus sequence in the A16 subunit of the entry–fusion complex, a furin inhibitor did not reduce virus entry unlike results obtained with a human papillomavirus control. These data support the use of this NanoBiT assay for studies of vaccinia virus entry, with application to basic research, antivirals, and neutralizing antibodies.

**IMPORTANCE:**

Assays to measure virus entry into cells mostly rely on post-entry events such as gene expression, cytopathic effects, or virus yields. Although fluorescence and electron microscopy can provide direct analyses, such methods are labor intensive and semiquantitative. Here we describe a rapid and quantitative real-time direct entry assay for vaccinia virus with application to basic research, antivirals, and neutralizing antibodies.

## INTRODUCTION

Assays to measure virus entry into cells are important for basic research and evaluation of antivirals and neutralizing antibodies. However, most assays rely on post-entry events such as gene expression, cytopathic effects, or virus yields. Although fluorescence and electron microscopy can provide direct analyses, such methods are labor intensive and semiquantitative. One applicable technology employs split luciferase fragments: a 1.3 kDa (11 amino acids) HiBiT and an 18 kDa LgBiT that can spontaneously reconstitute the active enzyme (1). To implement this method, the small HiBiT tag is genetically fused to the open reading frame of a virus protein, and LgBiT is expressed in the cytoplasm of a susceptible host cell. Luminescence is measured following the addition of a cell-permeable luciferase substrate. This approach has been used extensively with HiBiT-tagged virus-like particles and pseudoviruses (2–9) and with a recombinant virus (10). In the latter study, an infectious foot-and-mouth disease virus was constructed with the HiBiT peptide fused to the capsid protein VP1.

Poxviruses, of which vaccinia virus (VACV) is the prototype, are large DNA viruses that replicate exclusively in the cytoplasm (11). There are two main infectious forms of VACV: the intracellular mature virion (MV) consists of a core containing the genome and transcription apparatus, two proteinaceous lateral bodies, and a single outer lipoprotein membrane; the extracellular enveloped virion (EV) is essentially an MV surrounded by an additional double-layer membrane. The EV is important for exocytosis and virus spread, but the extra membranes must be disrupted, exposing the MV for fusion with cell membranes (12). *In vitro* studies show that MVs bind to cell surface glycosaminoglycans (8) and that entry is a two-step process involving hemifusion followed by pore formation (13), which is mediated by a complex of 11 conserved proteins that forms the entry–fusion complex (EFC) embedded in the MV membrane (14, 15). Fusion can occur at the plasma membrane or following endocytosis and is dependent on actin dynamics and activation of signaling pathways (16–18). The ability of VACV to infect a range of mammalian, avian, and insect cells suggests the absence of a unique cell receptor.

Recombinant VACVs with a reporter gene, such as firefly luciferase, have been used to monitor entry (16). However, with this system, transcription of the genome and translation of mRNA must occur before a signal is detected, which can take 40 min (13). The split luciferase direct entry method would be advantageous by eliciting a signal prior to and independent of the transcription and translation steps. To implement this approach, homologous recombination was used to generate a VACV (vA4-HiBiT) in which the native A4 (OPG130) protein was tagged with HiBiT at the C-terminus. A4 is an abundant 39 kDa core protein that is expressed during the late stages of infection, is modified by phosphorylation and O-GlcNAcylation, and is required for morphogenesis (19–22). The protein has long intrinsically disordered regions, a helical N-terminus associated with trimers of A10 (OPG136) that form the outer core wall, and a C-terminus that may associate with the lateral bodies or envelope (23–25). *In vitro* studies show that the MV membrane must be removed for the antibody to access A4 (26), and binding of the anti-A4 antibody to cores has been used in fluorescence-based entry assays (27). Here, we demonstrate the use of A4 in a split luciferase assay for analyzing the dynamics of VACV entry and the effects of neutralizing antibodies and pharmacological inhibitors.

## RESULTS

### Construction of a recombinant VACV expressing A4-HiBiT

**Fig 1:**
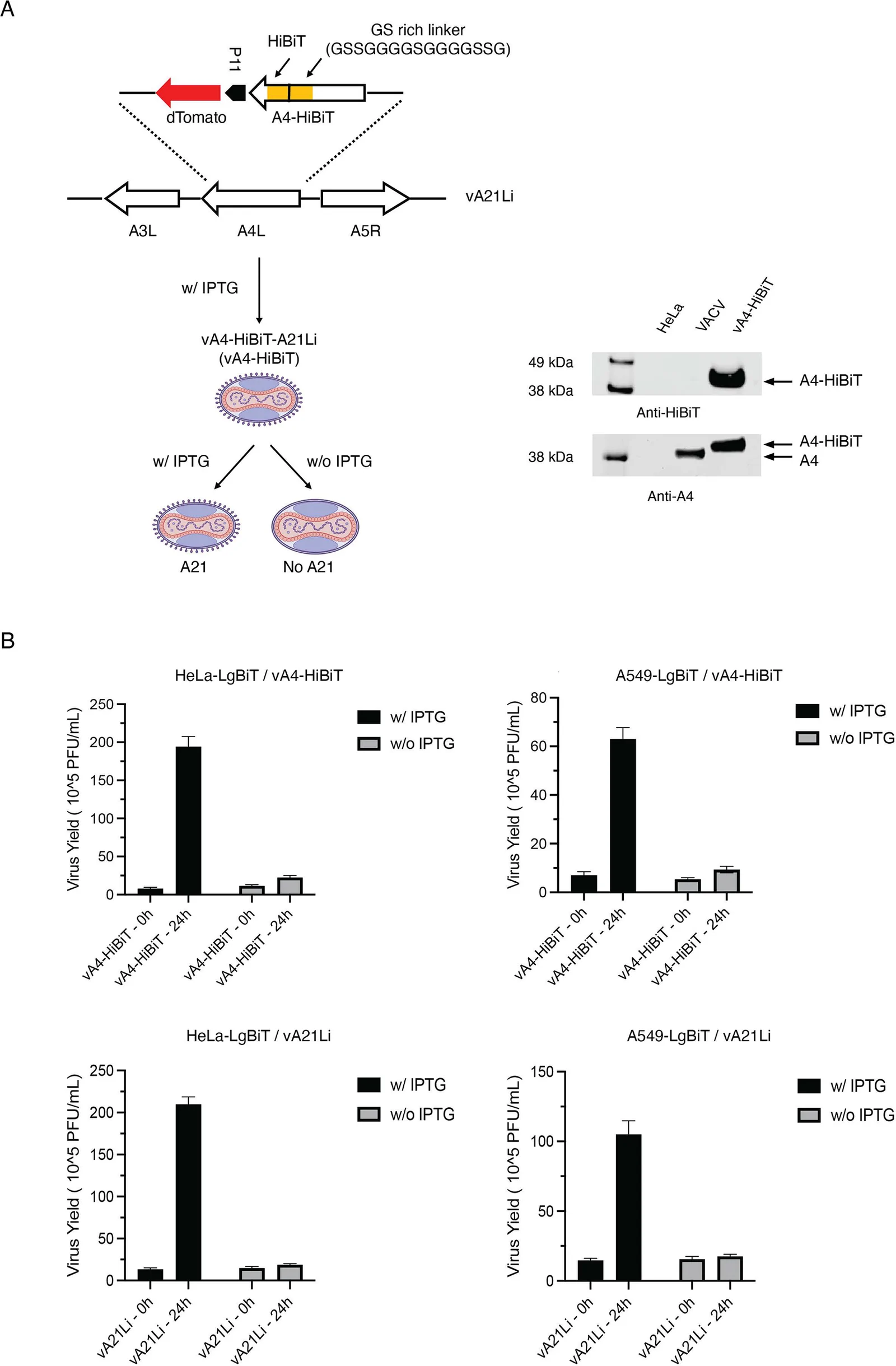
Construction and characterization of vA4-HiBiT. (**A**) Scheme showing the replacement of the A4L gene of vA21Li, a recombinant A21-inducible virus, with DNA containing HiBiT linked to the C-terminus of the A4L open reading frame through a flexible linker and the dTomato reporter gene. Following homologous recombination in the presence of IPTG (w/ IPTG), vA4-HiBiT-A21Li (abbreviated vA4-HiBiT) was isolated from fluorescent plaques. Virus with and without associated A21 was purified from cells infected w/ IPTG or without IPTG (w/o IPTG), respectively. Western blots of uninfected HeLa cells and cells infected with control VACV and vA4-HiBiT were probed with anti-HiBiT (upper blot) and anti-A4 (lower blot) antibodies. Upper blot: arrow points to the position of A4-HiBiT. Lower blot: arrows point to A4-HiBiT and A4. Positions of molecular weight markers are shown on the left. (**B**) Virus yields determined in triplicate at 0 and 24 h after infection of HeLa-LgBiT and A549-LgBiT cells with vA4-HiBiT in the presence or absence of IPTG or vA21Li. Data are shown as mean ± SEM.

To establish a NanoBiT-based protein–protein interaction platform for monitoring cell entry of VACV, homologous recombination was used to insert a modified A4L gene with HiBiT fused to the predicted C-terminus through a flexible linker in place of the corresponding native A4L gene of vA21Li to form vA4-HiBiT-A21Li (abbreviated vA4-HiBiT) as illustrated in Fig. 1A and Fig. S1A. vA21Li is a recombinant VACV with an IPTG-inducible A21L gene. A21 is 1 of 11 essential proteins that form the EFC (28, 29). When cells are infected with vA21Li in the absence of IPTG, virus particles are formed that lack the A21 protein and a functional EFC, as depicted in the diagram (Fig. 1A). Such viruses can bind to cells but not enter them. By using vA21Li as the parental virus to construct vA4-HiBiT, we confirmed that the split luciferase assay is measuring the entry step, as will be shown below.

Cells were infected with the vA4-HiBiT recombinant, which expresses the dTomato reporter, in the presence of IPTG, and plaques were screened for fluorescence. The virus was isolated from fluorescent plaques, and the process was repeated until all plaques were positive and only the modified gene could be detected by PCR. Synthesis of A4-HiBiT was demonstrated by Western blotting (Fig. 1A). Anti-HiBiT antibody stained a specific band of the predicted mass from purified vA4-HiBiT virions but not from control VACV. Furthermore, the A4-HiBiT protein detected by anti-A4 antibody migrated more slowly than native A4. These results confirmed the successful generation of vA4-HiBiT.

HeLa and A549 cells stably expressing LgBiT were used to complement vA4-HiBiT. To establish the compatibility of the virus and cells, HeLa-LgBiT and A549-LgBiT cells were infected with vA4-HiBiT or vA21Li in the presence and absence of IPTG and harvested at 0 and 24 h. In each case, virus yields were determined in BS-C-1 cells by plaque assay in the presence of IPTG. vA4-HiBiT titers were similar to those of vA21Li in HeLa-LgBiT cells and slightly lower in A549-LgBiT cells when infections were carried out with IPTG. Without IPTG, vA4HiBiT and vA21Li titers were very low in both cell lines (Fig. 1B), consistent with the slight leakiness of the system (28). These data indicate that vA4-HiBiT was successfully generated and retained A21-dependent virus production, providing a suitable assay system for evaluating VACV entry.

### Dependence of vA4-HiBiT entry on a functional EFC

**Fig 2:**
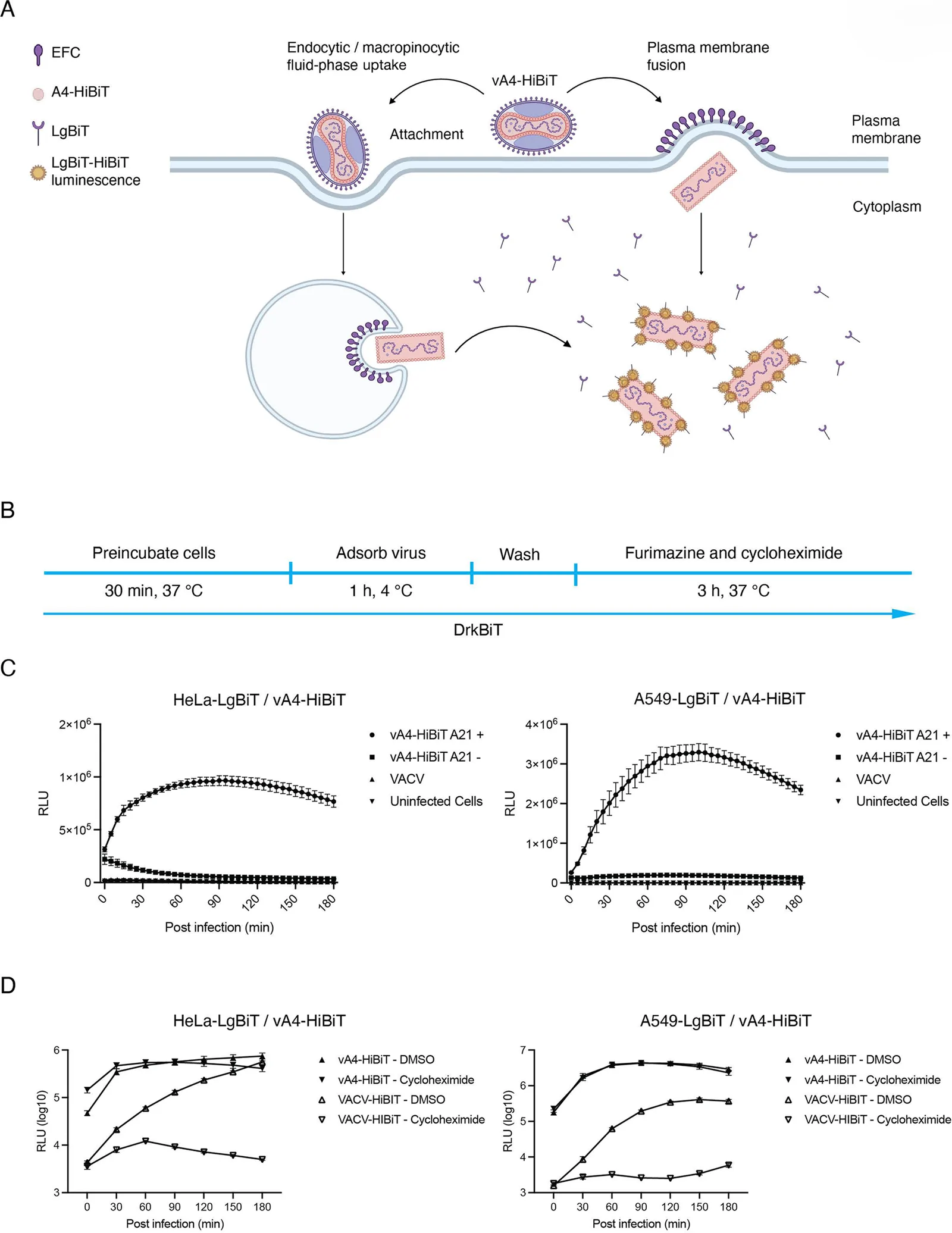
Rapid detection of entry is dependent on a functional EFC and independent of gene expression. (**A**) Depiction of vA4-HiBiT entry into cells expressing LgBiT via the endosomal or plasma membrane route. Following entry and core release into the cytoplasm, A4-HiBiT interacts with LgBiT to reconstitute enzymatically active NanoLuc luciferase. (**B**) Protocol timeline. Following a 30 min preincubation with DrkBiT at 37°C, HeLa-LgBiT and A549-LgBiT were incubated with the same number of genome copies of vA4-HiBiT produced in the presence (A21+) or absence (A21−) of IPTG, equivalent to 40 PFU/cell of A21+ virus for 1 h at 4°C. Wild-type VACV and uninfected cells served as negative controls. After virus adsorption, cells were washed and incubated with furimazine and cycloheximide. DrkBiT was maintained throughout to reduce background luminescence. (**C**) Rapid detection of VACV entry. Luminescence was measured immediately and every 5 min for 3 h at 37°C and plotted as relative light units (RLU). Infections were carried out in triplicate, and data are shown as mean ± SEM. (**D**) Entry of vA4-HiBiT is unaffected by an inhibitor of protein synthesis. HeLa-LgBiT and A549-LgBiT cells were infected in the presence of cycloheximide with vA4-HiBiT or a recombinant VACV (VACV-HiBiT) expressing HiBiT regulated by an early/late promoter. Luminescence was analyzed, and the RLU was calculated over a 3 h period. Infections were carried out in triplicate, and data are shown as mean ± SEM.

Virus entry by fusion at the plasma membrane or endocytic vesicle with delivery of the core into the cytoplasm is diagrammed in Fig. 2A. A4-HiBiT exposed at the surface of cytoplasmic cores can interact with LgBiT, resulting in luminescence upon addition of cell-permeable furimazine substrate. To evaluate the specificity of the assay, A21+ and A21− vA4-HiBiT virus particles were produced in the presence and absence of IPTG, respectively, and purified. HeLa-LgBiT and A549-LgBiT cells were preincubated with the membrane-impermeable DrkBiT peptide, which was maintained throughout the experiment, to compete with any extracellular LgBiT and reduce background (Fig. 2B). The vA4-HiBiT A21+, vA4-HiBiT A21−, and control VACV were incubated with cells at 4°C to synchronize virus binding. After washing, a 37°C medium containing the furimazine luciferase substrate and cycloheximide to prevent gene expression was added, and luminescence was monitored at 5 min intervals and plotted as relative light units (RLU). Luminescence was detected within the initial 5 min after the shift to 37°C with vA4-HiBiT A21+ and plateaued between 60 and 90 min before decreasing (Fig. 2C). In contrast, very low luminescence was detected with vA4-HiBiT A21− and near background with VACV or uninfected cells. The low luminescence produced by vA4-HiBiT A21− can be attributed to slight leakiness of the repression system. Similar results were observed with HeLa-LgBiT and A549-LgBiT cells, although the latter produced a higher peak signal. Together, these results demonstrate that the vA4-HiBiT assay can rapidly detect VACV entry and distinguish entry-competent virions with an intact EFC from entry-defective virions with a non-functional EFC. In all further experiments presented, only purified A21+ vA4-HiBiT was used.

### vA4-HiBiT entry is independent of gene expression

Some previous cell entry assays depend on gene expression. Here, we compared vA4-HiBiT with a VACV-HiBiT recombinant virus in which HiBiT expression is regulated by a viral early/late promoter instead of being incorporated into the virus particle. Cells were infected in the presence of cycloheximide or the equivalent amount of DMSO carrier, and luminescence was quantified over time (Fig. 2D). vA4-HiBiT generated a strong luminescence signal that plateaued by 60 min and was unaffected by cycloheximide. By contrast, VACV-HiBiT produced a delayed signal that increased gradually over time and was strongly inhibited by cycloheximide, consistent with dependence on early viral protein synthesis. Thus far, we showed that the vA4-HiBiT assay elicits a strong luminescence signal within 5 min, requires a functional EFC, and is independent of viral protein synthesis.

### Proportionality of luminescence to virus multiplicity

**Fig 3:**
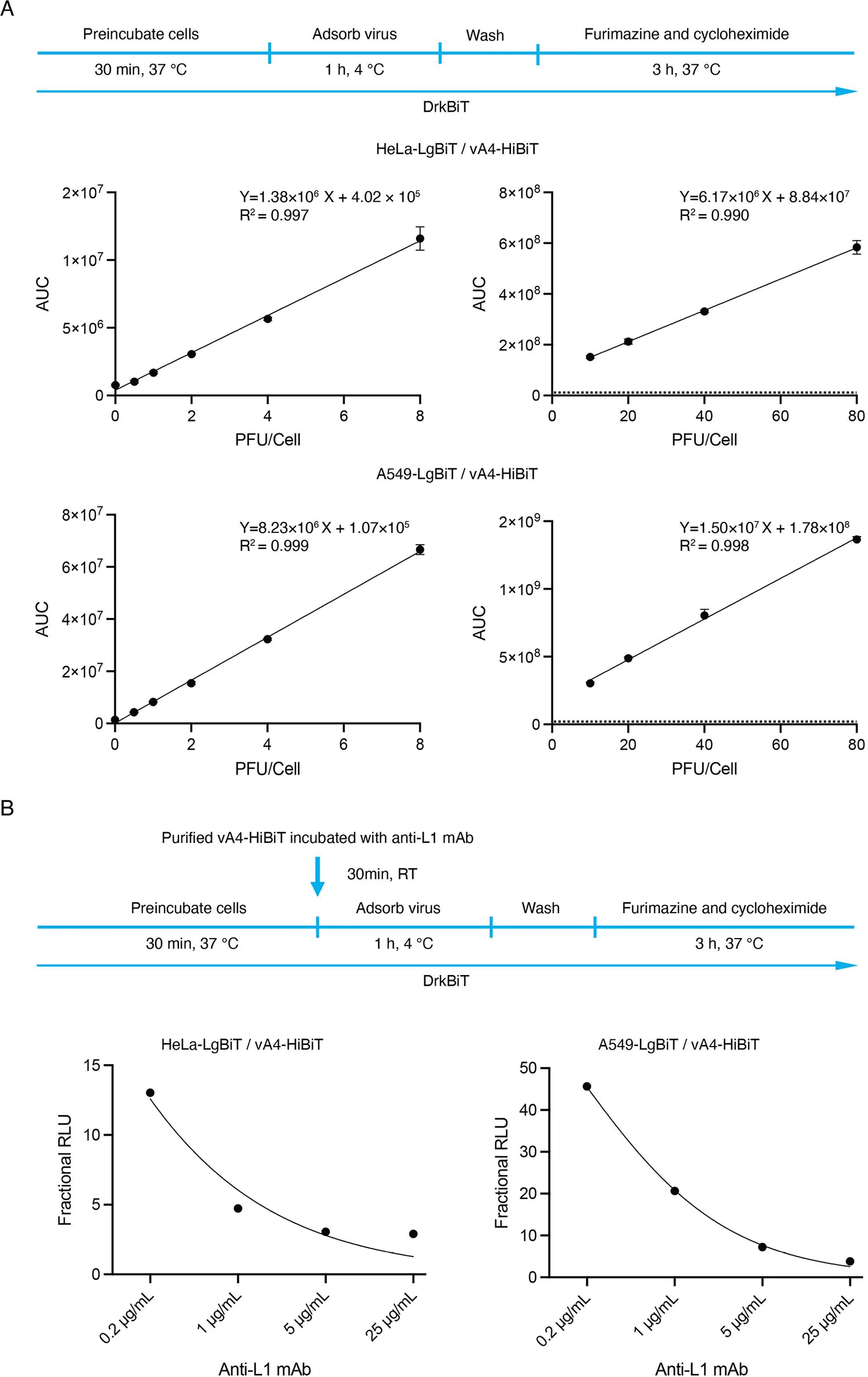
Luminescence signal is proportional to virus multiplicity and reduced by neutralizing antibody. (**A**) Correspondence of virus multiplicity and luminescence. Following preincubation with DrkBiT, HeLa-LgBiT and A549-LgBiT were incubated with 0 to 8 (left) or 10–80 (right) PFU/cell of vA4-HiBiT for 1 h at 4°C in triplicate. After virus adsorption, cells were washed and incubated with furimazine and cycloheximide at 37°C for 3 h. Luminescence signals were quantified by area under the curve (AUC) and analyzed by linear regression. The baseline signal determined from uninfected cells is indicated by the dashed line in the right panel. (**B**) Inhibition of vA4-HiBiT entry by neutralizing antibody. Purified vA4-HiBiT was incubated with the indicated concentrations of anti-L1 mAb prior to infection of HeLa-LgBiT or A549-LgBiT cells with a final multiplicity of 1 PFU/cell in triplicate. Luminescence was expressed as fractional RLU relative to the untreated vA4-HiBiT virus.

A similar protocol (Fig. 3A) was used to investigate the relationship between virus multiplicity and luminescence. HeLa-LgBiT and A549-LgBiT cells were infected with 0–8 PFU/cell and 10–80 PFU/cell of vA4-HiBiT, and area under the curve (AUC) values were calculated. A strong signal was detected with as little as 1 PFU/cell, and proportionality was maintained to at least 80 PFU/cell with high *R*^2^ values (Fig. 3A).

### Neutralizing antibody inhibits entry of vA4-HiBiT

Having established that the assay is proportional to virus multiplicity, we investigated the effect of an antibody that neutralizes infectivity. L1 is an EFC protein and a well-established target of neutralizing antibodies (30, 31). Purified vA4-HiBiT virions were preincubated with anti-L1 mAb for 30 min at room temperature and then added to HeLa-LgBiT or A549-LgBiT cells. Luminescence was measured after the addition of furimazine and cycloheximide and expressed as fractional RLU relative to the untreated vA4-HiBiT virus (Fig. 3B). Inhibition was detected at the lowest concentration of mAb tested and increased in a concentration-dependent manner in both HeLa-LgBiT and A549-LgBiT cells. The susceptibility of vA4-HiBiT entry indicates that the assay can be used for rapid screening of neutralizing antibodies.

### Comparison of luminescence substrates

**Fig 4:**
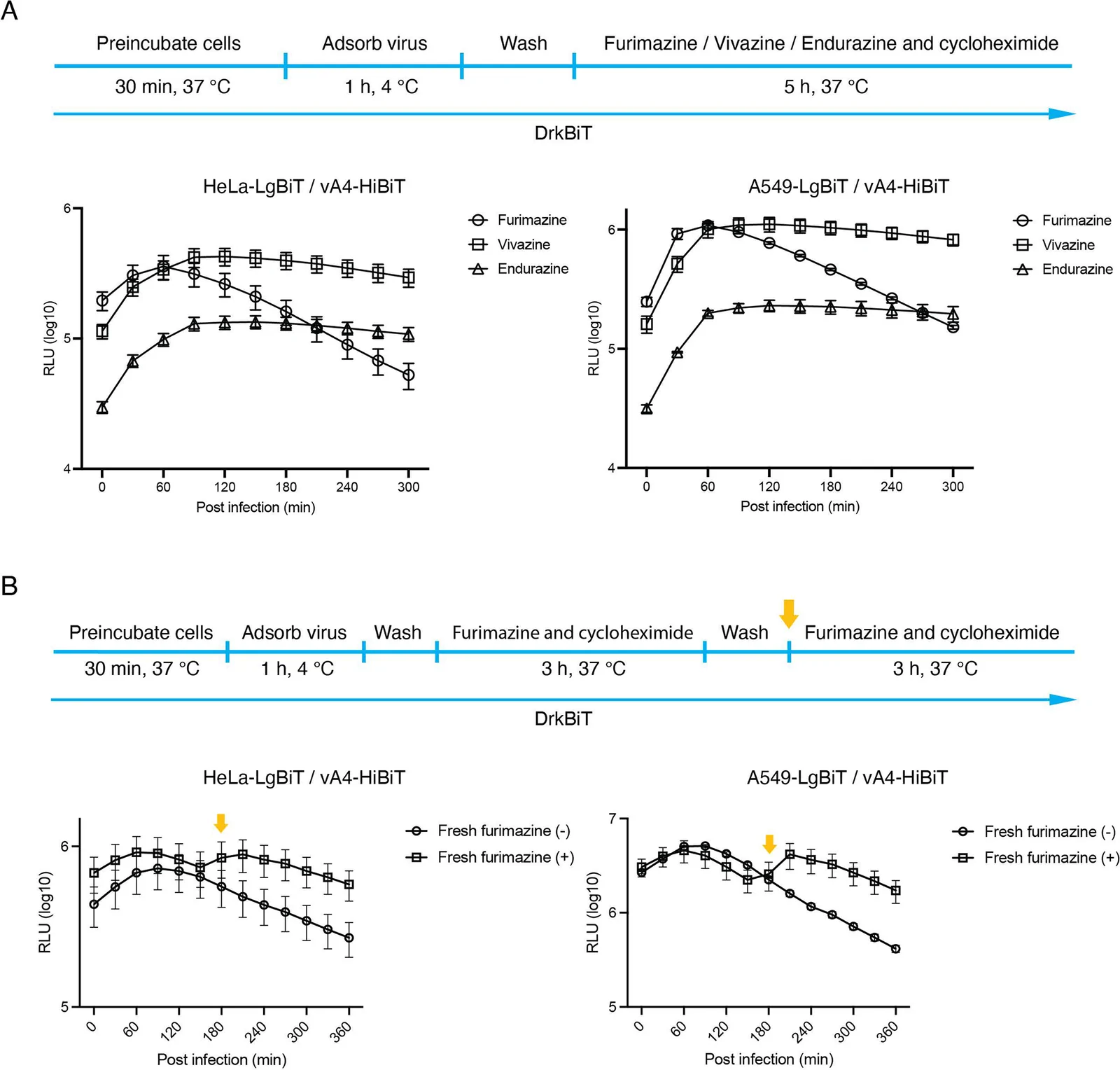
Comparison of luciferase substrates. (**A**) Assays with furimazine, vivazine, and endurazine. HeLa-LgBiT and A549-LgBiT cells were preincubated with DrkBiT for 30 min at 37°C and incubated with 40 PFU/cell of vA4-HiBiT for 1 h at 4°C in triplicate. After washing, cells were incubated at 37°C with cycloheximide and furimazine, vivazine, or endurazine. Luminescence was measured every 30 min for 5 h and plotted as RLU. (**B**) Effect of furimazine replenishment on luciferase activity. Following virus adsorption and washing, as in panel A, HeLa LgBiT and A549-LgBiT cells were incubated with furimazine and cycloheximide for 3 h at 37°C in triplicate. Cells were then washed, and fresh furimazine and cycloheximide were added to vA4-HiBiT (+), whereas no additional furimazine was added to vA4-HiBiT (−). Luminescence was monitored for an additional 3 h. Yellow arrows indicate the point at which fresh furimazine was added. Data are shown as mean ± SEM.

Three NanoLuc substrates are available: furimazine is a direct substrate with a relatively short half-life, whereas cell esterases slowly convert the other two substrates, vivazine and endurazine, to furimazine, thereby prolonging substrate availability. Furimazine provides the highest maximum intensity but the fastest signal decay; vivazine provides a more stable signal but lower maximum intensity, and the endurazine signal is the most stable but provides the lowest maximum intensity. The three substrates were compared in the vA4-HiBiT assay in Fig. 4A. The vivazine signal was slightly delayed relative to furimazine but was of similar peak magnitude and was maintained for a longer period. Endurazine generated the lowest luminescence signal but was very stable over time. Thus, furimazine and vivazine are most suitable for strongly detecting early entry-associated signals.

To confirm that the decrease in furimazine signal over time reflected substrate limitation rather than loss of HiBiT–LgBiT complementation, we performed a substrate replenishment experiment. Following virus adsorption at 4°C, cells were incubated with furimazine and cycloheximide at 37°C for 3 h. The cells were then washed; fresh furimazine and cycloheximide were added; and luminescence was monitored for an additional 3 h (Fig. 4B). Addition of fresh furimazine restored or increased the luminescence signal in both HeLa-LgBiT and A549-LgBiT cells, indicating that the substrate had been exhausted.

### VACV entry is modulated by pH and endosomal acidification

**Fig 5:**
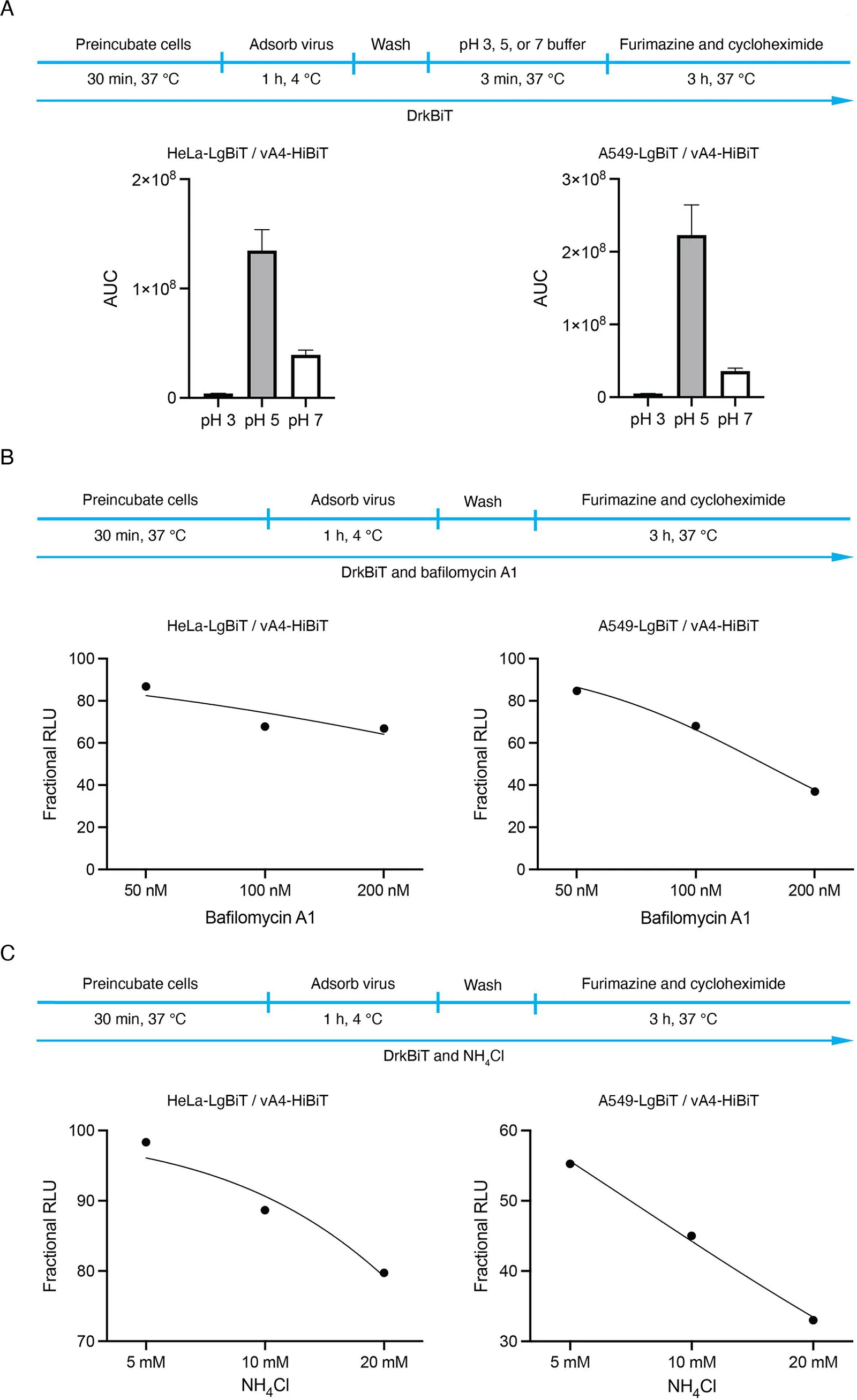
Effects of pH and inhibitors of endosomal acidification on entry. (**A**) Effect of brief pH treatment on entry. Following preincubation, vA4-HiBiT was adsorbed to HeLa-LgBiT and A549-LgBiT for 1 h at 4°C. After washing, the cells were incubated with pH 3, 5, or 7 buffer for 3 min at 37°C. The buffer was neutralized, and cells were incubated with furimazine and cycloheximide at 37°C for 3 h. Luminescence was quantified, and AUC was plotted. Data are shown as mean ± SEM. (**B**) Effect of bafilomycin A1 on entry. HeLa-LgBiT and A549-LgBiT were preincubated with the indicated concentrations of bafilomycin A1, which were maintained throughout the experiment. Following the preincubation, vA4-HiBiT was allowed to bind cells for 1 h at 4°C. After washing, the cells were incubated with furimazine and cycloheximide for 3 h at 37°C. Luminescence is plotted as fractional RLU relative to the equivalent concentrations of the DMSO carrier. (**C**) Effect of NH_4_Cl on entry. Procedures as in panel B, except that HeLa-LgBiT and A549-LgBiT cells were incubated with NH_4_Cl, and luminescence was plotted as fractional RLU relative to PBS. All assays were carried out in triplicate.

Subsequent experiments were designed to validate and extend previous studies that had been mainly carried out with indirect entry assays. The enhancement of VACV entry by brief low pH treatment and the reduction by inhibitors of endosome acidification suggest the importance of the endocytic pathway, although the magnitude of the effects varies with cell type and VACV strain (16, 32–36). In the present study, vA4-HiBiT bound to HeLa-LgBiT and A549-LgBiT cells at 4°C was exposed to pH 3, 5, or 7 buffers for 3 min at 37°C prior to measuring luminescence at neutral pH. The pH 5 treatment enhanced luminescence several-fold compared with pH 7 in HeLa-LgBiT cells and to a greater extent in A549-LgBiT cells. Brief pH 3 treatment almost completely reduced entry in both cell lines (Fig. 5A).

Next, we tested the effects of bafilomycin A1, an inhibitor of vacuolar ATPase-dependent endosomal acidification, on virus entry. HeLa-LgBiT and A549-LgBiT cells were pretreated with the drug, which was maintained through all subsequent steps. Bafilomycin A1 reduced the fractional RLU signal in a concentration-dependent manner, with a stronger inhibitory effect in A549-LgBiT than in HeLa-LgBiT cells (Fig. 5B). As bafilomycin A1 uptake could vary in the two cell lines, we also tested the effects of NH₄Cl, a lysosomotropic weak base that neutralizes acidic endosomal compartments. Cells were preincubated with NH₄Cl, and the compound was maintained throughout the assay and luminescence detection. NH₄Cl inhibited the A4-HiBiT signal in both HeLa-LgBiT and A549-LgBiT cells in a concentration-dependent manner (Fig. 5C) Like bafilomycin A1, entry was more strongly inhibited by NH_4_Cl in A549-LgBiT than HeLa-LgBiT cells.

### Actin dynamics are required for efficient entry

**Fig 6:**
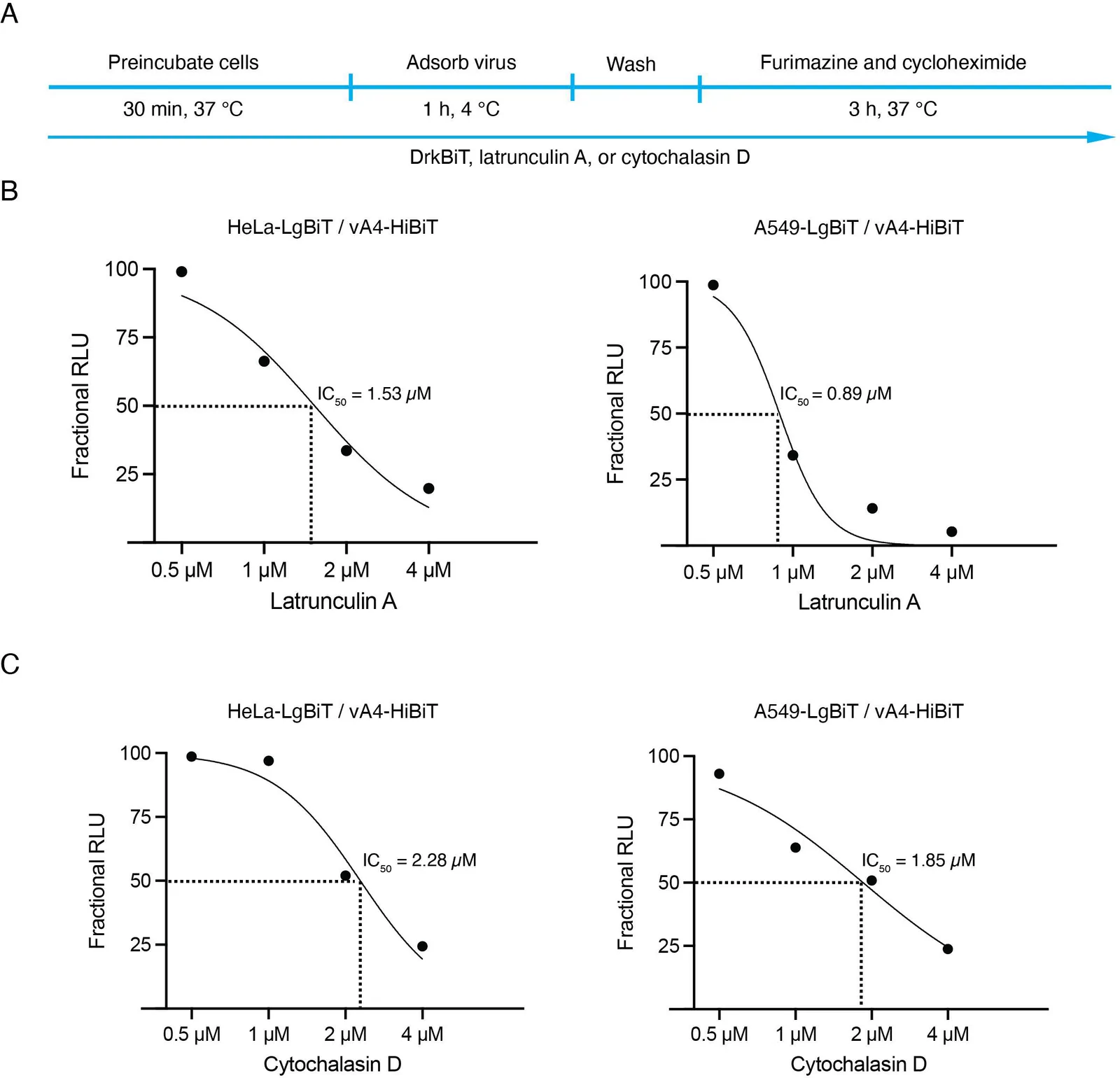
Effects of inhibitors of actin polymerization on VACV entry. (**A**) Experimental timeline. HeLa-LgBiT and A549-LgBiT cells were preincubated with DrkBiT and latrunculin A or cytochalasin D for 30 min at 37°C in triplicate. Cells were then incubated with vA4-HiBiT for 1 h at 4°C to synchronize virus adsorption. After washing, cells were incubated with furimazine and cycloheximide at 37°C for 3 h, and entry-associated luminescence was measured. Inhibitors were maintained throughout the experiments. (**B**) Effect of latrunculin A on VACV entry–fusion. Latrunculin A was added at concentrations of 0.5, 1, 2, and 4 µM in triplicate. Fractional RLU values were calculated relative to the equivalent concentrations of the DMSO carrier. (**C**) Effect of cytochalasin D on VACV entry–fusion. Cytochalasin D was added at concentrations of 0.5, 1, 2, and 4 µM in triplicate. Fractional RLU values were calculated relative to the DMSO control.

VACV entry has been reported to depend on actin dynamics, particularly during endocytic uptake (13, 33, 34). To support these results with the direct entry assay, we tested two actin inhibitors: latrunculin A and cytochalasin D. Latrunculin A disrupts actin polymerization by sequestering actin monomers, whereas cytochalasin D interferes with actin filament elongation. HeLa-LgBiT and A549-LgBiT cells were preincubated with the indicated concentrations of inhibitor and then infected with vA4-HiBiT in the presence of the drug (Fig. 6A). Latrunculin A inhibited VACV entry-associated luminescence in both HeLa-LgBiT and A549-LgBiT cells with IC_50_ values of approximately 1 µM (Fig. 6B).

In both HeLa-LgBiT and A549-LgBiT cells, cytochalasin D caused dose-dependent inhibition with IC_50_ values of approximately 2 µM (Fig. 6C). The inhibition observed with two mechanistically distinct compounds supports a role for actin dynamics in VACV entry and further validates the vA4-HiBiT assay as a platform for identifying cellular requirements for VACV entry.

### VACV entry is not dependent on furin activity

**Fig 7:**
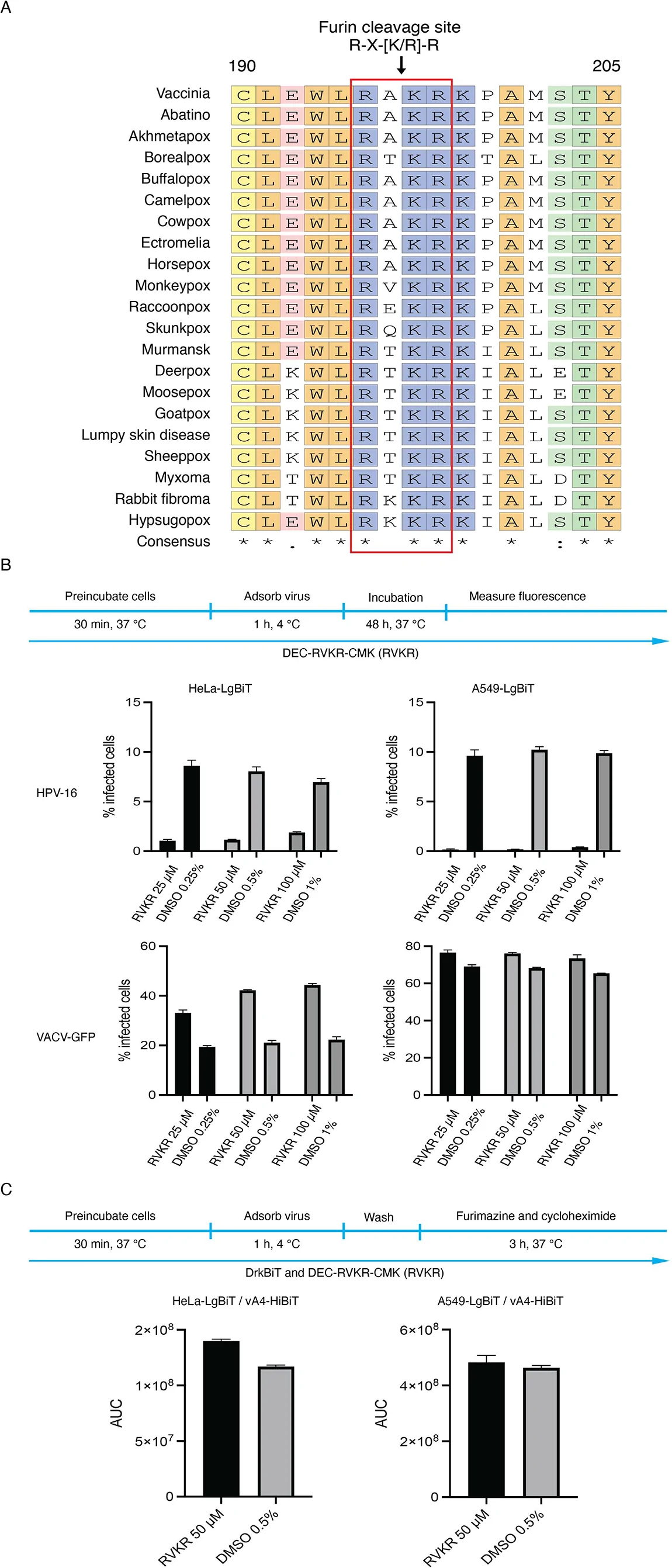
Inhibition of HPV but not VACV entry by a furin inhibitor. (**A**) Sequence alignment of A16 homologs from representative poxviruses. The R-X-[K/R]-R furin cleavage motif is aligned within the red outline. Sequences are shown for the following orthopoxviruses: VACV (AAA48348.2), Abatino (YP_010085803.1), Akhmetapox (QEQ49692.1), borealpox virus (QED21147.1), buffalopox virus (WCS73424.1), camelpox virus (NP_570524.1), cowpox virus (NP_619932.1), Ectromelia virus (NP_671638.1), horsepox virus (DAD53504.1), monkeypox virus (NP_536554.1), raccoonpox virus (YP_009143444.1), skunkpox virus (YP_009282829.1), and poxviruses of other genera: Murmansk poxvirus (YP_009408314.1), deerpox virus W-848-83 (YP_227492.1), moosepox virus GoldyGopher14 (AYC44788.1), goatpox virus (YCL82037.1), lumpy skin disease virus (AAN02833.1), sheeppox virus (XCY56265.1), myxoma virus (AGU99789.1), rabbit fibroma virus (NP_051995.1), and hypsugopox virus (QDJ95049.1). Nucleotide numbers are shown for VACV. (**B**) Effect of DEC-RVKR-CMK on HPV-16 and VACV entry. HeLa and A549 cells were preincubated with the furin inhibitor (abbreviated RVKR) or DMSO carrier for 30 min at 37°C in triplicate and then with HPV-16-GFP or VACV-GFP for 1 h at 4°C. Following adsorption, the cells were incubated at 37°C for 48 h. The percentages of GFP-positive cells were determined by flow cytometry. (**C**) Effect of DEC-RVKR-CMK on vA4-HiBiT entry. HeLa-LgBiT and A549-LgBiT cells were preincubated with DrkBiT and 50 µM DEC-RVKR-CMK or DMSO for 30 min at 37°C in triplicate, followed by vA4-HiBiT adsorption for 1 h at 4°C. After washing, cells were incubated with furimazine and cycloheximide for 3 h at 37°C, and luminescence was quantified as AUC. DEC-RVKR-CMK was maintained throughout the experiment.

Furin is a cellular proprotein convertase that is important for cell entry of many viruses including SARS-CoV-2, MERS-CoV, Ebola, Dengue, and HPV by cleaving envelope or capsid proteins to facilitate receptor binding or membrane fusion (37–40). However, there are no reports indicating whether furin is used for entry by poxviruses. Analysis of the 11 EFC proteins revealed the presence of the consensus furin cleavage motif Arg-X-Lys/Arg-Arg in the A16 protein, an essential component of the VACV EFC. Alignment with poxvirus A16 homologs showed that this motif is conserved in all orthopoxviruses as well as some members of other genera (Fig. 7A). We used decanoyl-RVKR-CMK (DEC-RVKR-CMK), an irreversible furin inhibitor, to assess whether furin activity is required for VACV entry. We confirmed that the drug inhibited HPV entry following a previously described protocol (40). The cells were preincubated with DEC-RVKR-CMK or a DMSO carrier and then infected with HPV-16 expressing green fluorescent protein (GFP). After 48 h, the percentage of GFP-positive cells was quantified by flow cytometry. DEC-RVKR-CMK drastically reduced HPV-16 infection in both cell lines at concentrations of 25 µM or higher compared to the equivalent amounts of DMSO carrier (Fig. 7B). Two methods were used to assess the effects of DEC-RVKR-CMK on VACV entry. The first method employed a recombinant VACV expressing GFP (41) and was carried out by a method similar to the one used for HPV-16. In contrast to the inhibitory effect on HPV, the percentage of GFP-expressing cells was increased with DEC-RVKR-CMK treatment (Fig. 7B).

The HiBiT/LgBiT assay was the second method used to assess a role for furin activity in VACV entry. HeLa-LgBiT and A549-LgBiT cells were treated with 50 µM DEC-RVKR-CMK, infected with vA4-HiBiT, and entry-associated luminescence was calculated as AUC. Furin inhibitor treatment had little to no effect on the A4-HiBiT signal in either cell line compared with the equivalent amounts of DMSO carrier (Fig. 7C). Despite the presence of a conserved furin cleavage motif, pharmacological inhibition of furin did not impair VACV entry.

## DISCUSSION

To implement the split luciferase assay, we tagged the A4 protein with HiBiT as A4 is positioned on the core surface where it can interact with cytoplasmic LgBiT upon removal of the MV membrane during penetration of the plasma or endosomal cell membrane. Importantly, the short 11-amino acid tag had little or no impact on virus infectivity. For this proof-of-principle study, we added the HiBiT tag to A4 in a virus with an inducible A21 protein to confirm that the assay is dependent upon a functional EFC. For future studies, wild type or other strains or mutants of VACV could be substituted, depending on the purpose of experiments. As A4 is highly conserved, we suspect that similar A4-HiBiT constructs could be made for other poxviruses to monitor entry. HeLa and A549 cells, which are commonly used for experiments with VACV, were stably transfected with HiBiT for this study.

vA4-HiBiT entry was unaffected by cycloheximide, a protein synthesis inhibitor, and could be detected within minutes, demonstrating that the assay is direct and not dependent on gene expression. The NanoLuc substrate furimazine was primarily used in this study. Although luminescence declined between 1 and 2 h, addition of fresh furimazine restored the peak signal, indicating that the decline was due to exhaustion of the substrate rather than reduction of the HiBiT–LgBiT interaction. Vivazine and endurazine are alternative substrates that provide a steady slow release of furimazine catalyzed by cellular esterases. Vivazine would be a good choice for most entry experiments, as the signal was detected nearly as fast and was more stable than with furimazine. Endurazine also provided signal stability, but the intensity was lower than with the other substrates.

Several earlier studies had shown that entry of VACV can be enhanced by brief low pH treatment that promotes fusion at the plasma membrane and reduced by inhibitors of endosomal acidification (16, 33, 42). Entry was increased by pH 5 treatment of vA4-HiBiT bound to either HeLa-LgBiT or A549-LgBiT cells, but the effect was stronger in the latter. Entry was severely inhibited by pH 3 treatment, as had been previously determined using a β-galactosidase expression assay (35). Using the HiBiT–LgBiT assay, we determined that pH 3 treatment directly inhibits entry.

Bafilomycin A1, an inhibitor of endosomal acidification, has been reported to inhibit infectivity to different extents, depending on the cell line and virus strain (16, 35, 43). Using vA4-HiBiT with the Western Reserve strain of VACV, there was greater inhibition in A549-LgBiT than HeLa-LgBiT cells. To consider whether this reflected the accessibility or activity of the drug to lower endosomal pH in different cell lines or relative use of neutral and low pH pathways for entry, the effect of NH_4_Cl was determined. Again, A549-LgBiT was more sensitive than HeLa-LgBiT. The greater enhancement provided by pH 5 treatment and the greater inhibition by bafilomycin A1 and NH_4_Cl treatments of A549-LgBiT compared to HeLa-LgBiT suggest differences in their relative use of neutral and low pH entry pathways. Little or no effect of NH_4_Cl as well as chloroquine on the entry of VACV MVs in RK13 cells was reported in an earlier study (44), although several inhibitors of endosomal acidification were shown in another report to have a strong negative effect on entry in RK13 cells (16).

Entry of VACV can be blocked by inhibitors of actin polymerization in HeLa cells (13, 33, 34). We found that latrunculin A and cytochalasin D inhibited entry into both HeLa-LgBiT and A549-LgBiT cells with IC_50_ values of approximately 1 and 2 µM, respectively.

Furin is a cellular proprotein convertase that operates mainly in the trans-Golgi network, endosomes, and cell surface and plays an important role in cell entry for many viruses, including SARS-CoV-2, MERS-CoV, Ebola, dengue, and HPV, by cleaving envelope or capsid proteins to facilitate receptor binding or membrane fusion (37–40). However, there were no reports indicating whether furin is used by poxviruses. Analysis of the 11 EFC proteins revealed the consensus furin cleavage motif Arg-X-Lys/Arg-Arg in the VACV A16 EFC protein as well as other orthopoxvirus A16 homologs. DEC-RVRK-CMK is an inhibitor of furin as well as six other proprotein convertases, namely, PC1, PC2, PC4, PACE4, PC5, and PC7. However, we found that entry of VACV into HeLa and A549 cells that had been treated with DEC-RVRK-CMK increased or was unaffected. It remains to be determined whether the consensus sequence in A16 is susceptible to cleavage and whether the motif is conserved for another function.

In summary, vA4-HiBiT provides a simple and rapid direct entry assay that can be used for VACV basic research and to determine the effect of inhibitors and neutralizing antibodies. Constraints of the method include the construction of recombinant viruses tagged with HiBiT and the generation of cell lines expressing LgBiT. In addition, the present study was carried out with the purified MV form of VACV.

## MATERIALS AND METHODS

### Cells and viruses

HeLa, A549, CV-1, and BS-C-1 cells were obtained from the ATCC and cultured in Eagle’s minimum essential medium supplemented with 10% fetal bovine serum. HPV-16-GFP was provided by Dr. Patricia M. Day (40). The VACV strain Western Reserve (WR) was propagated in HeLa cells, and all recombinant VACVs were derived from this strain.

### Construction of vA4-HiBiT

A PCR product was generated in which DNA encoding HiBiT was fused to the C terminus of the A4L open reading frame through a glycine-serine-rich linker (GSSGGGGSGGGGSSG). The construct also contained DNA encoding dTomato driven by the VACV P11 promoter downstream of A4L and was flanked by portions of the A3L and A5R genes. In-Fusion-compatible flanking sequences were incorporated at both termini by overlap-extension PCR using VACV WR genomic DNA as the template (Fig. S1A). The PCR product was inserted into a linearized pUC19 vector using the In-Fusion HD Cloning Kit (Takara Bio, USA) according to the manufacturer’s instructions. The final construct was confirmed by DNA sequencing. Generation of vA4-HiBiT by homologous recombination was adapted from a previously described protocol (22). Briefly, the recombinant plasmid was linearized with SacI and HindIII and transfected using Lipofectamine 2000 reagent (Thermo Fisher, Waltham, MA, USA) into BS-C-1 cells infected with vA21Li (28) in the presence of IPTG. Diluted lysates were applied to BS-C-1 cell monolayers, and plaques containing the recombinant virus were identified by fluorescence microscopy. Virus was recovered from fluorescent plaques, and plaque purification was repeated until clonal purity was achieved. Recombinant virus purity was confirmed by PCR, DNA sequencing, and immunoblotting.

### Construction of VACV-HiBiT

A 1,096-bp DNA fragment encoding the fusion protein mCherry–P2A–Ub–HiBiT was synthesized by Twist Bioscience. mCherry was fused in-frame to a porcine teschovirus 2A ribosome-skipping sequence, human ubiquitin, and the HiBiT peptide. In this design, 2A-mediated ribosomal skipping sequence separates mCherry from the Ub–HiBiT product during translation, and the ubiquitin–HiBiT junction is subsequently cotranslationally processed by ubiquitin carboxyl-terminal hydrolases to release HiBiT. The synthesized fragment was cloned downstream of the VACV p7.5 promoter in pSC11 (45) that had been linearized with SalI and NotI, using NEBuilder Master Mix (New England Biolabs). CV-1 cells grown in six-well plates were infected with wild-type VACV and subsequently transfected with 2 μg of the donor plasmid using Lipofectamine 2000 (Thermo Fisher) to promote homologous recombination. The recombinant virus was plaque-purified three times on the basis of red fluorescence and BrUdR resistance (46).

### Construction of HeLa and A549 cells that express LgBiT

A549 and HeLa cells stably expressing LgBiT were generated using the Sleeping Beauty transposase system (47, 48). The codon-optimized LgBiT sequence synthesized by IDT was cloned into the pSBbi-BH vector using the *SfiI* restriction site (Fig. S1B). The resulting pSBbi-BH LgBiT plasmid was cotransfected with the transposase-expressing plasmid pCMV(CAT)T7-SB100 in a 10:1 molar ratio into A549 and HeLa cells using TransIT-LT1 (Mirus Bio). Forty-eight hours after transfection, the cells were passaged and selected with 250 µg/mL Hygromycin B Gold (InvivoGen) for 2 weeks. The successful integration of the transposable cassette was confirmed by expression of TagBFP in flow cytometry 2–4 weeks after selection and by functional assay using Nano-Glo HiBiT Lytic Detection System (Promega, N3040). The cells were maintained in the complete medium with 250 µg/mL of Hygromycin B Gold. pSBbi-BH was a gift from Eric Kowarz (Addgene plasmid #60515; https://www.addgene.org/60515/, RRID:Addgene_60515). pCMV(CAT)T7-SB100 was a gift from Zsuzsanna Izsvak (Addgene plasmid #34879; https://www.addgene.org/34879/, RRID:Addgene_34879).

### Virus purification

VACV and recombinant viruses were grown in HeLa cells in the presence or absence of IPTG, and MVs were purified by ultracentrifugation through a 36% (wt/vol) sucrose cushion, followed by banding through 24%–40% sucrose gradients. Purified virus was pelleted and resuspended in 1 mM Tris-Cl, pH 9.0, aliquoted, and stored at −80°C. Infectivity was determined by plaque assay on BS-C-1 cells (46) and genome copies by droplet digital PCR.

### Quantification of viral genome copies by droplet digital PCR

vA4-HiBiT virions produced in the presence or absence of IPTG were purified by sucrose cushion and gradient centrifugation. Viral DNA was extracted using the DNeasy Blood and Tissue Kit (QIAGEN) according to the manufacturer’s protocol and treated with benzonase. DNA was serially diluted and analyzed by droplet digital PCR (ddPCR) using primers targeting the E11 gene (49) and QX200 ddPCR EvaGreen Supermix (Bio-Rad), following a previously described protocol (50). After 40 reaction cycles, droplets were analyzed using a Bio-Rad droplet reader, and absolute DNA copy numbers were determined.

### Protein extraction

Cells were lysed on ice in RIPA buffer containing 10 mM Tris-Cl, pH 7.5, 150 mM NaCl, 0.5 mM EDTA, 0.1% SDS, 1% Triton X-100, and 1% deoxycholate, supplemented with protease and phosphatase inhibitor cocktail (Sigma) and DNase I (Thermo Fisher). Protein concentrations were determined using the Qubit Protein BR Assay Kit (Invitrogen).

### Immunoblotting

Solubilized proteins were resolved by SDS-PAGE in 4%–12% gels and transferred to nitrocellulose membranes using iBlot 2 NC stacks (Invitrogen). Membranes were blocked with 5% non-fat milk and incubated with primary antibodies overnight at 4°C. After washing, membranes were incubated with IRDye-conjugated secondary antibodies (LI-COR) for visualization. Primary antibodies included anti-A4 antibody (51) and anti-HiBiT antibody (N7200, Promega).

### NanoBiT-based VACV entry assay procedure

A549-LgBiT and HeLa-LgBiT cells were seeded in the same opaque 96-well plate, and infections were carried out in triplicate. The following day, cells were preincubated with DrkBiT (1 µM) for 30 min at 37°C and then infected with vA4-HiBiT or VACV-HiBiT for 1 h at 4°C to synchronize virus adsorption. Unless otherwise indicated, all experiments used purified vA4-HiBiT virions produced in the presence of 50 µM IPTG. For a typical experiment, 40 PFU/cell of vA4-HiBiT produced in the presence of IPTG or an equivalent number of genome copies of vA4-HiBiT produced in the absence of IPTG were incubated with HeLa-LgBiT or A549-LgBiT cells for 1 h at 4°C. The cells were washed three times with PBS. Furimazine, prepared according to the manufacturer’s protocol, and cycloheximide (300 µg/mL) were then added, and luminescence was measured at the indicated times for up to 3 h at 37°C. DrkBiT (1 µM) was maintained throughout the experiment. Cycloheximide (C7698-1G) was purchased from Sigma-Aldrich and prepared as a 15 mM stock solution in DMSO. The Nano-Glo live cell assay system containing furimazine (N2012), the Nano-Glo endurazine and vivazine live cell substrates (N2590), and DrkBiT peptide (N7400) were from Promega.

### Flow cytometry entry assay procedure

HeLa and A549 cells were preincubated with the furin inhibitor or the equivalent amount of DMSO carrier for 30 min at 37°C in triplicate and then with HPV-16-GFP (20 ng) or VACV-GFP (2.5 × 10^4^ PFU/cell in the presence of 44 µg/mL AraC to prevent VACV replication and spread) for 1 h at 4°C and then at 37°C for 48 h. The percentages of GFP-positive cells were determined by flow cytometry.

### Inhibition of entry by mAb

Purified vA4-HiBiT was incubated with 0.2–25 µg of anti-L1 mAb (30) in 0.05% bovine serum albumin for 30 min prior to infection of HeLa-LgBiT or A549-LgBiT cells with a final multiplicity of 1 PFU/cell in triplicate. The cells were subsequently incubated with furimazine and cycloheximide at 37°C, and luminescence was measured for up to 3 h.

### Brief pH treatment

vA4-HiBiT was adsorbed to HeLa-LgBiT and A549-LgBiT for 1 h at 4°C in triplicate. After washing, the cells were incubated with pH 3 or 5 citrate saline buffer or pH 7 PBS for 3 min at 37°C. The buffer was neutralized with medium; the cells were incubated with furimazine and cycloheximide at 37°C; and luminescence was measured for up to 3 h.

### Pharmacological inhibitors

Inhibitors were incubated with cells for 1 h at 4°C and maintained throughout all subsequent steps. After virus adsorption, the cells were washed and incubated with furimazine and cycloheximide for 3 h at 37°C. Luminescence was determined as fractional RLU relative to the equivalent amounts of carrier. Bafilomycin A1 (HY-100558, MedChemExpress) was prepared as a 10 µM stock solution in DMSO and used at 50, 100, or 200 nM. Cytochalasin D (HY-N6682, MedChemExpress) was prepared as a 10 mM stock solution in DMSO and used at 0.5, 1, 2, and 4 µM. Latrunculin A (HY-16929, MedChemExpress) was prepared as a 237 µM stock solution in ethanol and used at 0.5, 1, 2, and 4 µM. NH₄Cl was dissolved in PBS to prepare a 2.5 M stock solution and used at 5, 10, and 20 mM. DEC-RVKR-CMK (344930-1MG, Sigma-Aldrich) was prepared as a 10 mM stock solution in DMSO and used at 25, 50, and 100 µM.

### Reproducibility and statistical analysis

All experimental observations, including virus yield measurements, were confirmed by independent experiments performed in triplicate. Data are presented as means, with error bars representing the standard error of the mean. Statistical analyses and AUC calculations were performed using GraphPad Prism 10. Fractional RLU was calculated by normalizing the RLU value of each treatment condition to the corresponding control condition using the following formula: fractional RLU = RLU_treatment_ / RLU_control_.

## Data Availability

All data supporting the findings of this study are available within the paper or will be provided upon request. Requested materials developed at NIH will be provided by B. Moss (bmoss@nih.gov) upon signing an NIAID material transfer agreement and assurance that work will be done under appropriate biosafety conditions.

## SUPPLEMENTAL MATERIAL

Fig. S1 jvi.01115-26-s0001.pdf Nucleotide sequences inserted into plasmids for construction of vA4HiBiT and for Lg-BiT-expressing cells.

ASM does not own the copyrights to Supplemental Material that may be linked to, or accessed through, an article. The authors have granted ASM a non-exclusive, world-wide license to publish the Supplemental Material files. Please contact the corresponding author directly for reuse.
