# Supplementary material for: Real-time tracking of vaccinia virus entry using a split luciferase complementation assay
Source: J Virol. 2026 Sep 29;100(10):e01115-26. doi: 10.1128/jvi.01115-26 (PMC13712968; doi:10.1128/jvi.01115-26)
Supplement: Supplementary file 1 [file jvi.01115-26-s0001.pdf]

A

5’ - In-Fusion site -Partial A3L flanking-dTomato-p11 Promoter-HiBiT-GS rich linker-A4L-Partial A5R flanking-In-Fusion site – 3’

5’CGGTACCCGGGGATCGCCCCTGCGGATATAAAGTCATCGTCTACAATTTGGGACAATGAATTGCATACACTACAA
GACAAAGATTTATCAGAAGTGTGAATATGATCTTCATCTACCAAAGAAAGAGTTTGATTAGTATAACTAGATTTTAGT
CCTGCGTTAGATGTTAAAAAAACATCGCTATTGACCACGGCTTC**CAT**TATTTATATTCGTAGTTTTTACTCGAAAGCG
TGATTTTAATATCCAATCTTA**TTA**CTTGTACAGCTCGTCCATGCCGTACAGGAACAGGTGGTGGCGGCCCTCGGAG
CGCTCGTACTGTTCCACGATGGTGTAGTCCTCGTTGTGGGAGGTGATGTCCAGCTTGGTGTCCACGTAGTAGTAG
CCGGGCAGTTGCACGGGCTTCTTGGCCATGTAGATGGTCTTGAACTCCACCAGGTAGTGGCCGCCGTCCTTCAG
CTTCAGGGCCTGGTGGATCTCGCCCTTCAGCACGCCGTCGCGGGGGTACAGGCGCTCGGTGGAGGCCTCCCA
GCCCATTGTCTTCTTCTGCATTACGGGGCCGTCGGGGGGGAAGTTGGTGCCGCGCATCTTCACCTTGTAGATCA
GCGTGCCGTCCTGCAGGGAGGAGTCCTGGGTCACGGTCACCAGACCGCCGTCCTCGAAGTTCATCACGCGCTC
CCACTTGAAGCCCTCGGGGAAGGACAGCTTCTTGTAATCGGGGATGTCGGCGGGGTGCTTCACGTACGCCTTG
GAGCCGTACATGAACTGGGGGGACAGGATGTCCCAGGCGAAGGGCAGGGGGCCGCCCTTGGTCACCTTCAGC
TTGGCGGTCTGGGTGCCCTCGTAGGGGCGGCCCTCGCCCTCGCCCTCGATCTCGAACTCATGGCCGTTCATGG
AGCCCTCCATGCGCACCTTGAAGCGCATGAACTCTTTGATGACCTCCTCGCCCTTGCTCAA**CAT**TTATAGCATAGA
AAAAAACAAAATGAAATT**TTA**AGAGATCTTCTTGAATAGTCTCCATCCAGAGACACCTGACGACCCTCCACCTCCG
CTCCCGCCACCACCGCTCGAGCCCTTTTGGAATCGTTCAAAACCTTTGACTAGTTGTAGAATTTGATCTATTGCCC
TACGCGTATACTCCCTTGCATCATATACGTTCGTCACCAGATCGTTTGTTTCGGCCTGAAGTTGGTGCATATCTCTT
TCAACATTCGACATGAGATCCTTAAGGGCCATATCGTCTAGATTTTGTTGAGATGCTGCTCCTGGATTTGGATTTTG
TTGTGCTGTTGTACATACTGTACCACCAGTAGGTGTAGGAGTACATACAGTGGCCACAATAGGAGGTTGAGGAGG
TGTAACCGTTGGAGTAGTACAAGAAATATTTCCATCCGATTGTTGTGTACATGTAGTTGTTGGTAACGTCTGAGAAG
GTTGGGTAGATGGCGGTGTCGTCGTCTTTTGATCTTTATTAAATTTAGAGATAATATCCTGAACAGCATTGCTCGGC
GTCAACGCTGGAAGGAGTGAACTCGCCGGCGCATCAGTATCTTCAGACAGCCAATCAAAAAGATTAGACATATCA
GATGATGTATTAGTTTGTTGTCGTGGTTTTGGTGTAGGAACAGTACTACTAGGTAGAAGAATAGGAGCCGGTGTAG
CTGTTGGAACCGGCTGTGGAGTTATATGAATAGTTGGTTGTAGCGGTTGGATAGGCTGTCTGCTGGCGACCGTCA
TATTATCTCTAGCTAGTTGTTCTCGCAACTGTCTTTGATAATACGACTCTTGAGACTTTAGTCCTATTTCAATCGCTT
CATCCTTTTTCGTATCCGGATCCTTTTCTTCAGAATAATAGATTGACGACTTTGGTGTAGAGGATTCTGCCAGCCCC
TGTGAGAACTTGTTAAAGAAGTC**CAT**TTAAGGCTTTAAAATTGAATTGCGATTATAAGA**TTA**AATGGCAGACACAGA
CGATATTATCGACTATGAATCCGATGATCTCACCGAATACGAGGATGATGAAGAAGAGGAAGAAGATGGAGAGTCA
CTAGAAACTAGTGATATAGATCCCAAATCTTCTTATAAGATTGTAGAATCAGCATCCACTCATATAGAAGATGCGCATT
CCAATCTTAAACATATAGGGAATCATATATCTGCTCTTAAACGACGCTATACTAGACGGATCCTCTAGAGTCG 3’

B

GGCCTCTGAGGCCACC**ATG**GTCTTCACACTCGAAGATTTCGTTGGGGACTGGGAACAGACAGCCGCCTACAACC
TGGACCAAGTCCTTGAACAGGGAGGTGTGTCCAGTTTGCTGCAGAATCTCGCCGTGTCCGTAACTCCGATCCAA
AGGATTGTCCGGAGCGGTGAAAATGCCCTGAAGATCGACATCCATGTCATCATCCCGTATGAAGGTCTGAGCGCC
GACCAAATGGCCCAGATCGAAGAGGTGTTTAAGGTGGTGTACCCTGTGGATGATCATCACTTTAAGGTGATCCTG
CCCTATGGCACACTGGTAATCGACGGGGTTACGCCGAACATGCTGAACTATTTCGGACGGCCGTATGAAGGCATC
GCCGTGTTCGACGGCAAAAAGATCACTGTAACAGGGACCCTGTGGAACGGCAACAAAATTATCGACGAGCGCCT
GATCACCCCCGACGGCTCCATGCTGTTCCGAGTAACCATCAACAGC**TAA**GGCCTGTCAGGCC

Fig. S1. Nucleotide sequences inserted into plasmids for construction of vA4HiBiT and for Lg-BiT expressing cells. **(A)** vA4HiBiT. In-fusion cloning was used to insert the DNA fragment into pUC19, which was transfected into cells infected with vA21LI for homologous recombination. Annotated sequences are color coded. **(B)** LgBiT expressing cells. The LgBiT DNA fragment was inserted into pSBbi-BH at the underlined SfiI recognition sites. The resulting plasmid was co-transfected into A549 and HeLa cells with a transposase expressing plasmid to generate HeLa-LgBiT and A549-LgBiT cells.
